# Genistein Improves Neuropathology and Corrects Behaviour in a Mouse Model of Neurodegenerative Metabolic Disease

**DOI:** 10.1371/journal.pone.0014192

**Published:** 2010-12-01

**Authors:** Marcelina Malinowska, Fiona L. Wilkinson, Kia J. Langford-Smith, Alex Langford-Smith, Jillian R. Brown, Brett E. Crawford, Marie T. Vanier, Grzegorz Grynkiewicz, Rob F. Wynn, J. Ed Wraith, Grzegorz Wegrzyn, Brian W. Bigger

**Affiliations:** 1 Mucopolysaccharidosis (MPS) Stem Cell Research Group, Biomedicine, Faculty of Medical and Human Sciences, University of Manchester, Manchester, United Kingdom; 2 Department of Molecular Biology, Faculty of Biology, University of Gdansk, Gdansk, Poland; 3 Zacharon Pharmaceuticals Inc., San Diego, California, United States of America; 4 Institut National de la Santé et de la Recherche Médicale (INSERM) Unit 820, Lyon University, Lyon, France; 5 Pharmaceutical Research Institute, Warsaw, Poland; 6 Bone Marrow Transplant Unit, Royal Manchester Children's Hospital, Manchester Academic Health Science Centre, Central Manchester University Hospitals NHS Foundation Trust, Manchester, United Kingdom; 7 Genetic Medicine, St. Mary's Hospital, Manchester Academic Health Science Centre, Central Manchester University Hospitals NHS Foundation Trust, Manchester, United Kingdom; Hungarian Academy of Sciences, Hungary

## Abstract

**Background:**

Neurodegenerative metabolic disorders such as mucopolysaccharidosis IIIB (MPSIIIB or Sanfilippo disease) accumulate undegraded substrates in the brain and are often unresponsive to enzyme replacement treatments due to the impermeability of the blood brain barrier to enzyme. MPSIIIB is characterised by behavioural difficulties, cognitive and later motor decline, with death in the second decade of life. Most of these neurodegenerative lysosomal storage diseases lack effective treatments. We recently described significant reductions of accumulated heparan sulphate substrate in liver of a mouse model of MPSIIIB using the tyrosine kinase inhibitor genistein.

**Methodology/Principal Findings:**

We report here that high doses of genistein aglycone, given continuously over a 9 month period to MPSIIIB mice, significantly reduce lysosomal storage, heparan sulphate substrate and neuroinflammation in the cerebral cortex and hippocampus, resulting in correction of the behavioural defects observed. Improvements in synaptic vesicle protein expression and secondary storage in the cerebral cortex were also observed.

**Conclusions/Significance:**

Genistein may prove useful as a substrate reduction agent to delay clinical onset of MPSIIIB and, due to its multimodal action, may provide a treatment adjunct for several other neurodegenerative metabolic diseases.

## Introduction

There are over 50 described lysosomal storage disorders (LSDs), affecting approximately 1/7000 live births [1]. Many of these are caused by defects in lysosomal enzyme function, leading to the accumulation of uncatabolised substrates, often resulting in progressive neurodegeneration, neuroinflammation and death in childhood [2]. Enzyme replacement therapies are limited to attenuated LSDs or those affecting visceral organs alone due to an inability of lysosomal enzymes to traffic across the adult blood brain barrier [3]. Haematopoietic stem cell transplantation is an efficient treatment for a very small subset of these disorders [4], but substrate reduction therapy (SRT) which relies on inhibition of substrate anabolism, or substrate clearance via alternative catabolic pathways, is emerging as an effective alternative for some glycosphingolipid LSDs [5]. SRTs are limited by the lack of agents able to effectively reduce substrate without significant toxic side effects.

The tyrosine kinase inhibitor genistein aglycone [6] reduces glycosaminoglycan (GAG) substrate accumulation in fibroblasts of several mucopolysaccharide LSDs [7] has low oral toxicity in mammals [8], [9] and around 10% blood brain barrier permeability [10]. Genistein in a supplement form has been given to patients with MPSIIIA and IIIB, GAG storing LSDs with no effective treatments [11], at 5 mg/kg/day, but efficacy, although encouraging, remains unclear [12]. We have recently shown that short-term administration of genistein significantly reduces liver lysosomal storage in mice with MPSIIIB [13]. Genistein has also been shown to inhibit lipopolysaccharide (LPS) induced TNF-alpha, IL1-alpha and IL6 production in mixed glial and astrocytic cultures [14] and inhibit microglial activation in mixed neuron-glial and microglial enriched cultures [15], suggesting a possible role in attenuating neuroinflammation. We tested the hypothesis that high doses of genistein given long-term could reduce brain storage of primary GAG substrates, secondary glycosphingolipids and reduce neuroinflammation, a common feature of many neurodegenerative diseases.

## Results

### Genistein reduces lysosomal size and storage of heparan sulphate in brain and liver

**Figure 1:**
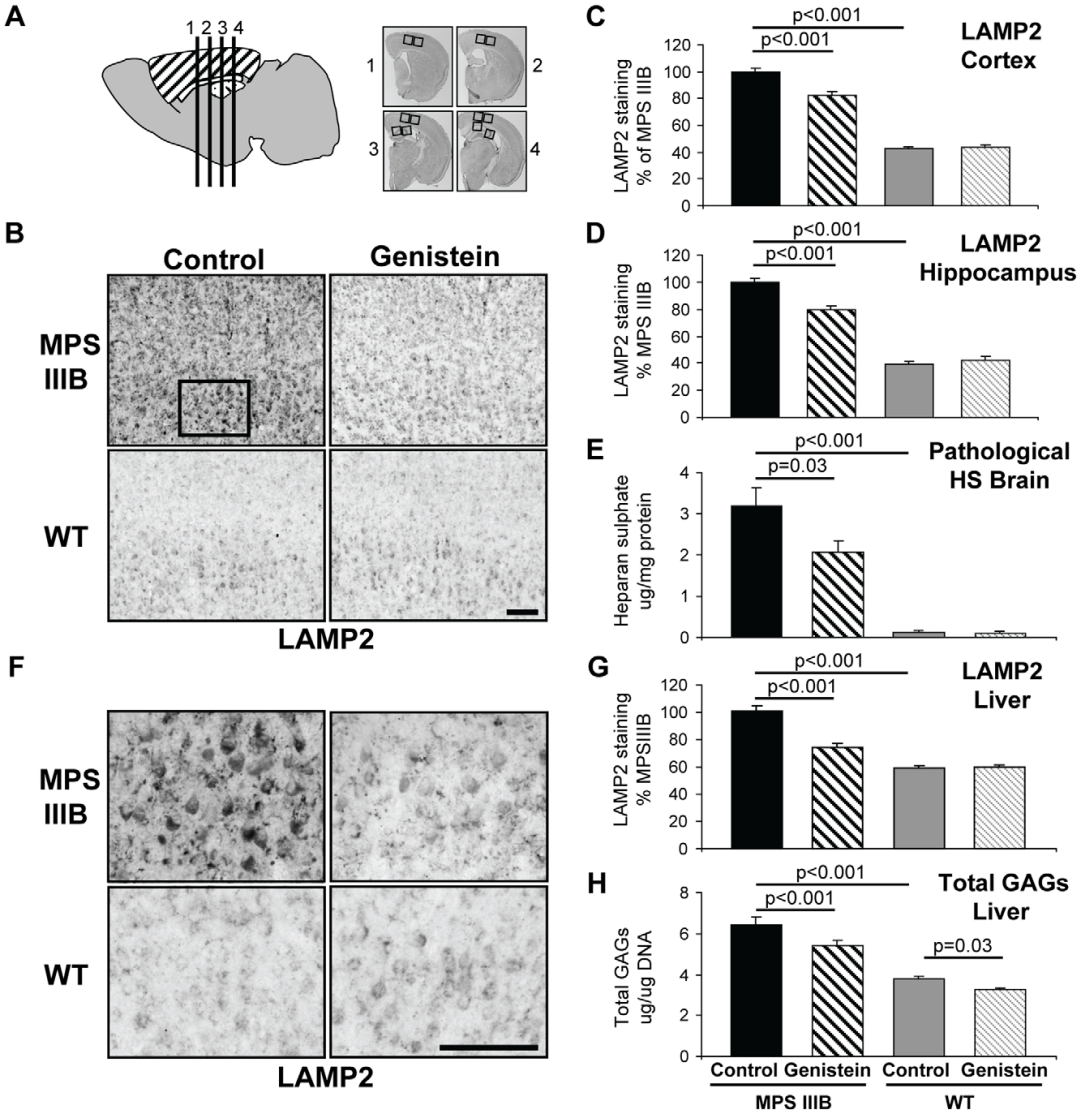
Primary storage substrates are reduced in brains of MPSIIIB mice after genistein treatment. (**A**) 11 month old MPSIIIB and WT, male and female mice with and without long-term genistein treatment were sacrificed and 30 µm coronal sections (numbered 1–4) were cut from each mouse at positions 0.26, −0.46, −1.18 and −1.94 relative to bregma. For cerebral cortex (hatched), two low power fields of view for each section (boxed) were quantified or positive cells counted (8 fields total), whilst for hippocampus (spots), two low power fields from sections 3–4 (boxed) were quantified (4 fields total). All sections were stained together and blinded to ensure consistency. (**B**) Representative Lysosomal Associated Membrane Protein (LAMP2) staining of cerebral cortex of 11 month old MPSIIIB and WT, male and female mice with and without long-term genistein treatment. This indicates the size of the lysosomal compartment and hence stored material in cells in layers II/III-V/VI of the cerebral cortex. The images correspond to section 2 shown in Figure 1A. Bar  = 100 µm. (**C**) Quantification of mean LAMP2 staining in cerebral cortex is expressed as a percentage of staining in untreated MPSIIIB mice. (**D**) Quantification of mean LAMP2 staining of hippocampus. (**E**) Mean weight of pathological heparan sulphate in the brain per mg protein, measured using the SensiPro assay. (**F**) High power view of cerebral cortex layer V - box from (**B**). Bar  = 100 µm. (**G**) Quantification of mean LAMP2 staining of 2 fields of view from 3 liver sections (6 fields total) is expressed as a percentage of staining in untreated MPSIIIB mice. (**H**) Mean weight of total glycosaminoglycans in the liver per µg DNA, measured using the Blyscan assay. For all graphs genders were pooled, thus n = 12 mice per group, error bars represent SEM, p values are for Tukey's multiple comparisons test.

MPSIIIB and control wild-type (WT) mice of both sexes were treated from 8 weeks of age for 9 months with a soy free diet or diet containing 160 mg/kg/day of genistein aglycone. Four coronal sections from each brain were stained and two fields of view for each section quantified (Figure 1A). Cells throughout the brains of MPSIIIB mice have an enlarged lysosomal compartment size as measured by the intensity of LAMP2 (lysosomal associated membrane protein 2) staining [16], and increased storage of the GAG, heparan sulphate. Following genistein treatment, highly significant 31% reductions in LAMP2 staining were observed in the cortex (Figure 1B,C,F), 34% in the hippocampus (Figure 1D) as well as a significant 37% reduction in the pathogenic heparan sulphate stored in the brains of MPSIIIB mice (Figure 1E). No changes in LAMP2 or heparan sulphate were seen in WT mice.

LAMP2 staining and total GAGs were also significantly reduced by 64% and 35% respectively in the livers of MPSIIIB mice receiving genistein (Figure 1G,H), whilst genistein treated WT mice showed significantly decreased liver GAGs (Figure 1H).

### Genistein reduces neuroinflammation in MPSIIIB mice

**Figure 2:**
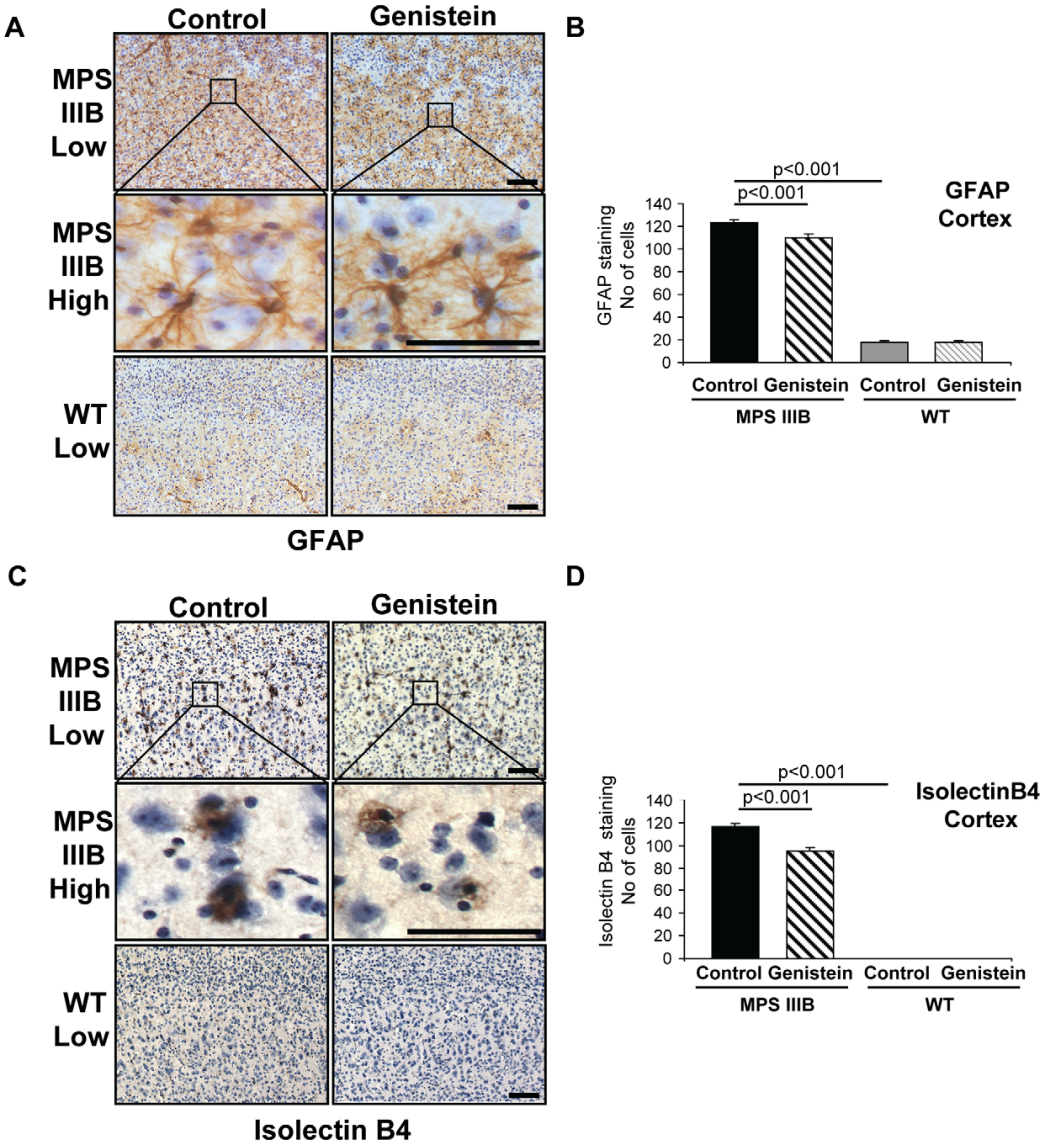
Neuroinflammatory markers are reduced in brains of MPSIIIB mice after genistein treatment. (**A**) Representative Glial Fibrillary Associated Protein (GFAP) staining of astrocytes (brown) in the cerebral cortex of 11 month old MPSIIIB and WT mice with and without long-term genistein treatment. Sections have been counterstained with haematoxylin to highlight nuclei (blue). The images correspond to section 2 shown in Figure 1A. Boxed areas are enlarged to show individual astrocyte cell bodies. Bar  = 100 µm for low power images and 50 µm for enlargements. (**B**) The mean number of GFAP positive cells in the cerebral cortex per low power field of view were counted as described in Figure 1. (**C**) Representative Isolectin B4 staining of microglial cells (brown) in the cerebral cortex. Sections have been counterstained with haematoxylin to highlight nuclei (blue). The images correspond to section 2 shown in Figure 1A. Boxed areas are enlarged to show individual microglial cells. Bar  = 100 µm for low power images and 50 µm for enlargements. (**D**) The mean number of Isolectin B4 positive cells in the cerebral cortex per low power field of view were counted. For all graphs genders were pooled, thus n = 12 mice per group, error bars represent SEM, p values are for Tukey's multiple comparisons test.

To determine if genistein could reduce neuroinflammation in MPSIIIB, we counted the number of GFAP-positive astrocytes (Figure 2A,B) and Isolectin B4-positive microglial cells (Figure 2C,D) in the cerebral cortex. MPSIIIB mice exhibit a marked increase in neuroinflammatory astrocytes and microglial cells [13], [16] concomitant with activation of several neuroinflammatory mediators [17], [18], [19] compared to WTs. Genistein significantly reduced both GFAP-positive astrocytes (12%) and Isolectin B4-positive microglial cells (19%) in the cerebral cortex of MPSIIIB mice whilst no change was seen in WT mice (Figure 2A–D). Furthermore, some microglia in the genistein treated MPSIIIB mice appear to be smaller and less intensely stained suggesting that they are less activated than microglia in untreated MPSIIIB mice.

### Genistein may reduce secondary metabolites and improves synaptic function

**Figure 3:**
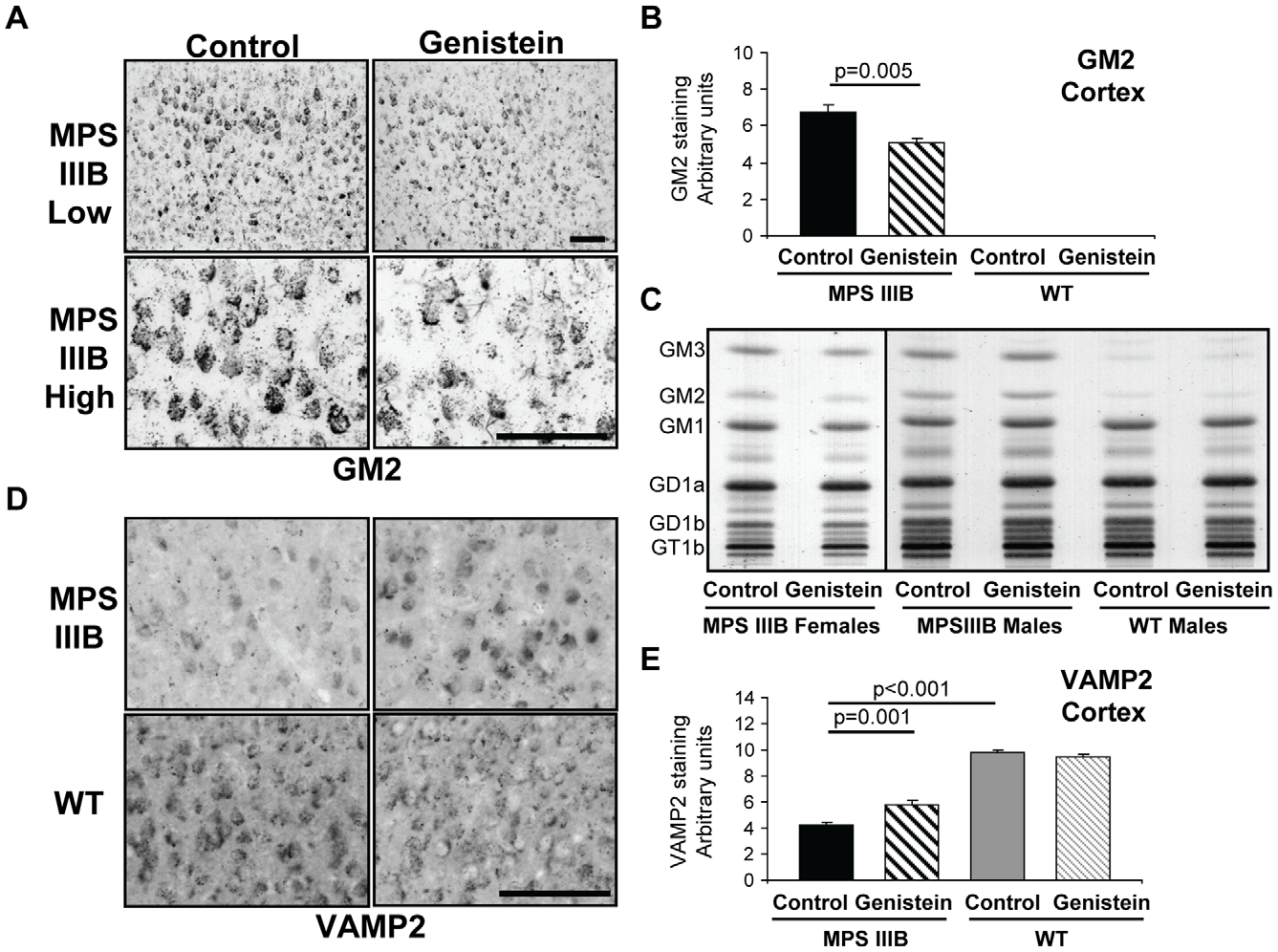
Some improvements were seen in secondary metabolite storage and markers of synaptic transmission in brains of MPSIIIB mice after genistein treatment. (A) Representative GM2 ganglioside staining in the cerebral cortex of 11 month old MPSIIIB mice with and without long-term genistein treatment at low (layers II/III-V/VI) and high power (layer V). The images correspond to section 2 shown in Figure 1A. Bar  = 100 µm. WT mice have no GM2 staining in the cortex and are not shown. (B) Quantification of mean GM2 staining of cerebral cortex in arbitrary units. (C) Chromatographic profiles of total gangliosides from the fourth brain hemicoronal fifth (rostral to caudal). Each lane represents 3mg wet weight of pooled sections from 4 mice. (D) Representative Vesicle Associated Membrane Protein (VAMP2) staining in the cerebral cortex. VAMP2 is a synaptic vesicle marker required for effective synaptic transmission. The images correspond to section 2 shown in Figure 1A. Bar  = 100 µm. (E) Quantification of mean VAMP2 staining of cerebral cortex in arbitrary units. For all graphs genders were pooled, thus n = 12 mice per group, error bars represent SEM, p values are for Tukey's multiple comparisons test.

In many LSDs, including MPSIIIB, secondary metabolites such as GM2 and GM3 gangliosides, as well as cholesterol, are accumulated [20], [21] as a result of the primary catabolic block. Immunohistochemistry showed significant GM2 ganglioside storage, particularly in layer II, III and V of the cortex in MPSIIIB mice that was significantly reduced by genistein (25%) (Figure 3A,B). In WT mice, GM2 was virtually undetectable in the cortex as previously shown [20]. The proportion of GM2 and GM3 measured biochemically and expressed as percentage of total gangliosides also showed clear pathologic elevation in untreated MPSIIIB mice (Figure 3C). However a minor reduction of GM2 was only observed in genistein treated MPSIIIB female mice (6.2% vs 7.4±0.5%) while GM3 remained unchanged in all mutants (12.5±0.1%). Because pooled brain sections were used for analysis, we cannot be confident that this small GM2 reduction is mediated by genistein. Histology reflects cerebral cortical GM2 between 0.26 to −1.94 mm relative to bregma, whilst biochemistry reflects ganglioside storage from −2.5 to −4.5 mm relative to bregma.

We observed a significant reduction of the pre-synaptic vesicle associated membrane protein, VAMP2 in the cerebral cortex (Figure 3D,E) and hippocampus (not shown) of MPSIIIB mice in agreement with our previous findings [16]. VAMP2 is part of the SNAP/SNARE complex involved in synaptic transmission, the loss of which has been shown to result in a dramatic reduction in synaptic function [22]. Genistein significantly improved VAMP2 staining in cortex but not in hippocampus of MPSIIIB mice.

### Genistein corrects behaviour of MPSIIIB mice

**Figure 4:**
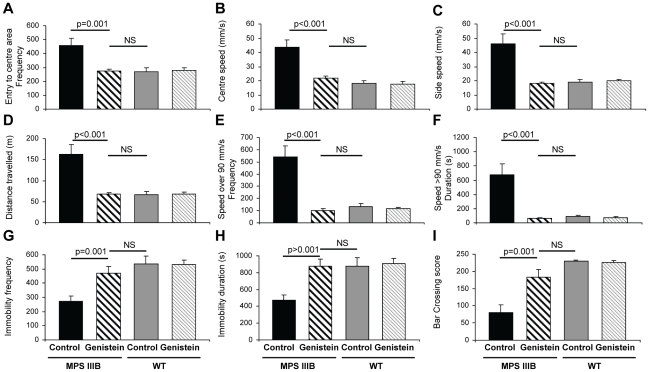
Genistein treatment corrects behavioural abnormalities seen in MPSIIIB mice. (**A**) 8 month old MPSIIIB and WT, male and female mice with and without long-term genistein treatment were monitored in the open field test of locomotor and exploratory activity for 60 minutes. The arena was divided into 12 squares and the frequency of entry (and total duration- not shown) to the central 4 squares measured to determine responses to danger and thigmotaxis. (**B**) The average speed in the central area and (**C**) side squares was also measured, as was (**D**) total distance travelled, to give an indication of abnormal locomotor activity. (**E**) Rapid exploratory behaviour was measured by frequency and (**F**) duration of speed more than 90 mm/s. (**G**) The number of times and (**H**) the duration of time spent immobile was also measured as speed under 0.05 mm/s. (**I**) Latency to cross or fall from a hanging bar is a measure of locomotor activity with some element of cognitive function due to a training period and was tested at 10 months of age. Significant gender*genotype effects were seen in centre and side speed, distance travelled, frequency of speed over 90 mm/s, immobility frequency and duration reflecting greater pathological effect in female MPSIIIB mice. For all graphs genders were pooled, thus n = 12–14 mice per group, error bars represent SEM, p values are for Tukey's multiple comparisons test.

Locomotor activity, anxiety and exploratory behaviour were monitored automatically in the open field test [23] over a 1 hour period, as well as frequency and duration of very rapid exploratory behaviour (speed>90 mm/s) and immobility (speed<0.05 mm/s) at 8 months of age. MPSIIIB mice showed a highly significant increase in the frequency with which they cross into or out of a central area (Figure 4A), their speed in this area or the side area (Figure 4B,C), the total distance travelled (Figure 4D), and frequency and duration of travelling at more than 90 mm/s (Figure 4E,F), indicating increased exploration. All of these parameters were fully normalised by genistein treatment. MPSIIIB mice showed significantly reduced frequency (Figure 4G) and duration of immobility (Figure 4H) which were also normalised by genistein treatment.

Significant gender*genotype effects were observed in centre and side speed, distance travelled, frequency of speed over 90 mm/s, immobility frequency and duration. Untreated female MPSIIIB mice performed significantly worse than male MPSIIIB mice on these tasks, however when genders were analysed separately, significant correction of MPSIIIB mice by genistein was observed in all cases except total distance travelled (p = 0.06) and frequency of immobility (p = 0.07) for untreated vs treated male MPSIIIB mice.

The hanging bar test measures motor co-ordination and has an element of memory and learning due to a training period [24]. MPSIIIB mice performed significantly worse than WT mice at 10 months (Figure 4I). In contrast, genistein treated MPSIIIB mice were completely corrected at 10 months and could not be distinguished from WT mice suggesting retention of motor function.

## Discussion

We have shown a significant reduction in lysosomal size in the cerebral cortex and hippocampus and in total brain storage of pathological heparan sulphate in MPSIIIB mice treated with genistein aglycone for 9 months. The fact that WT mice do not show a reduction in brain substrate and the lack of any obvious toxicity, suggests that genistein at these doses in the brain only weakly inhibits tyrosine kinases and this is borne out by chronic studies in the rat and dog suggesting low oral toxicity [8], [9].

We did see liver GAG reductions in WT mice treated with genistein which could reflect higher bioavailability in the liver [10], coupled with the high metabolic activity of this organ. We found a significant gender effect in total liver GAGs as observed previously [13], with male mice storing more GAGs than females. Genistein still significantly reduced storage in male and female MPSIIIB mice. No gender or gender*genotype effects were seen in the brain. Gender specific differences in GAG storage have not been reported to our knowledge in patients with MPSIIIB and may just reflect metabolic gender differences in inbred murine strains.

Our observation that genistein was able to reduce the number of microglial and astrocytic cells in MPSIIIB mice could have a dual explanation. Genistein has been reported to reduce LPS induced inflammatory cytokine production [14], and to inhibit microglial activation [15] *in vitro*, but it would be difficult to separate a role for genistein in direct modulation of neuroinflammation from that of reduced heparan sulphate storage, as reduction of heparan sulphate oligosaccharides ameliorates inflammation, although the converse may not be true [25]. Despite this apparent disconnect, further investigation of the role of genistein in neuroinflammation in other neurodegenerative diseases is warranted, since anti-inflammatories have been shown to reduce microglial cells in mouse models of Alzheimer disease [26], [27] and reduce neuroinflammation and prolong life in models of Sandhoff disease [28]. Thus although a reduction in inflammation is unlikely to reduce primary storage, it may help to improve behavioural manifestations of disease.

There was some evidence to suggest that genistein was also able to change other downstream neuropathological events. Although it was unclear if genistein was able to mediate changes in minor monosialogangliosides, the data was more clear cut for improvements in synaptic organisation as shown by VAMP2 staining [16]. This is unlikely to reflect a gain in function, instead it is more likely that genistein is able to delay loss of synaptic transmission, which would be important if this effect can be replicated in other neurodegenerative diseases.

Finally, behaviour of MPSIIIB mice was fully corrected to WT levels by genistein treatment. The hyperactive and increased exploratory locomotor behaviour of MPSIIIB mice is similar to that of MPSIIIB patients and is consistent with changes reported previously by us and others [16], [23]. We found the 60 minute open field test to be an equally good measure of abnormal locomotor activity in these mice as our circadian locomotor test, with the advantage of being much shorter [16], [23]. Interestingly, the duration spent in the centre of the cage was virtually unchanged in MPSIIIB or WT mice in our hands (not shown) suggesting a normal prey response to danger in these mice. This is in contrast to decreased responses to danger in MPSIIIB mice observed in the elevated plus maze [23], although the small size of our arenas could have reduced these responses [29] and may explain why we did not observe these differences. Males also showed reduced responses compared to females in several of these locomotor tests. MPSIIIB mice start to retain urine at 4–6 months of age (not shown), often leading to hydronephrosis or uremia, which may reflect autonomic control of urinary sphincter function or more likely, blockage of the urinary tract [30]. This manifestation of disease appears earlier in males than females, leads to gait abnormalities and is often the humane endpoint. This may limit male MPSIIIB mice in their locomotor activity giving the illusion of less pathology in these tasks. Given that urinary retention is not reported in humans with MPSIIIB, extended studies of lifespan in MPSIIIB mice where urinary retention is the primary endpoint have limited value in indicating improvements in neuropathology.

Genistein is widely available as a food supplement, occurring naturally within soy foods predominantly in a glycoside form (genistin) [31] and can also be synthesised or purified in its aglycone form. A concentrated form of soy extract has been used in clinical trial for patients with MPSIIIA and IIIB at 5–10 mg/kg/day, doses that we would predict from our previous data [13] would not be effective in the brain but may clear peripheral storage. This open label study was not designed to study neurological outcomes but did show small reductions in urinary GAGs [12]. Although genistein has been widely tested for safety and efficacy at reducing incidence of various forms of cancer [32] its only approved clinical indication to our knowledge is for osteopenia using relatively low doses [33]. Our use of genistein aglycone, which is reported to be less susceptible to degradation by gut flora than genistin [31], has high plasma bioavailability [34] and delivery of high doses to ensure sufficient blood brain barrier diffusion [10] may explain why our approach was effective.

In conclusion, genistein aglycone significantly reduces brain lysosomal storage, and neuroinflammation, delays synaptic loss and corrects behaviour in mice with MPSIIIB. Due to its multimodal actions, genistein may prove applicable in delaying clinical onset of disease and neuroinflammation in MPSIIIB and similar neurodegenerative metabolic diseases.

## Materials and Methods

### Mouse maintenance and drug administration

Animal procedures were ethically approved and carried out in accordance with UK Home Office regulations under project licence PPL 40/3056. The MPSIIIB knock-out mouse [35] was maintained as a heterozygote line on an inbred C57BL/6J background at the University of Manchester, UK as previously described [16]. Eight week old male and female MPSIIIB and WT mice (n = 6–7 per group) received a soy free diet (2014 Teklad Global Rodent Diet, Harlan, England) or the same diet containing 160 mg/kg/day genistein aglycone (Pharmaceutical Research Institute, Warsaw, Poland). 2 mice from the MPSIIIB untreated and 2 from the MPSIIIB treated groups were found dead at 9–10 months of age and excluded from biochemical or histological analysis.

### Behavioural testing

All animals had access *ad libitum* to food and water. Behavioural testing was performed between 8:00 and 10:00 A.M. on naïve mice, standardizing as many environmental parameters as possible [29]. The tester was blinded to genotype.

### Open field test

8 month old mice were placed in the centre of one of 4 opaque arenas (480×375×210 mm) and monitored for 60 minutes by digital camera. Arenas were cleaned between sessions. Videos were analyzed and scored in an unbiased fashion using TopScan Suite (Clever Sys. Inc, Virginia, USA) and random videos were checked for consistency of automated scoring. Locomotor measures included total distance travelled, duration and frequency of immobility (speed less than 0.05 mm/s), duration and frequency of rapid exploration (speed over 60 mm/s and 90 mm/s). The arena was divided into 12 squares and frequency and duration of crossover to or from the 4 central squares was used to measure locomotor activity and response to danger. Frequency and duration of rearing was not found to be consistent using Topscan suite.

### Hanging bar test

Mice were tested individually with randomization. The test was modified from [24]. Briefly, a metal bar 25 cm long was located horizontally between 2 wooden columns. The animal was held by the tail and allowed to grasp the centre of the bar with forepaws only before release. Latency to cross or fall was scored as described [24]. Each animal had 3 training trials with a 10 min rest before the final scored trial. Results are presented as an average of 3 trials, and the test was performed at 10 months of age.

### Tissue preparation

Mice were sacrificed by dislocation of the cervical vertebrae. Brains were divided into two hemispheres. One hemisphere was fixed (4% paraformaldehyde/PBS) for 24 hours followed by cryoprotection with 30%sucrose/PBS/2mMgCl_2_ for 48 hours at 4°C and stored at −80°C for histological analysis. The entire fixed hemisphere was cut into coronal sections (30 µm) using a sledge microtome (Hyrax S30, Zeiss, Germany) and each section stored in sequential wells of a 96 well plate. Staining was performed on free-floating sections before mounting on superfrost slides (Fisher Scientific, USA). The other hemisphere was divided into hemicoronal fifths, snap frozen, and stored at −80°C for biochemistry. For liver, half a lobe was fixed (4% paraformaldehyde/PBS) and embedded in paraffin for histology. 6 µm-thick sections were mounted on superfrost slides before staining. The other half was snap frozen for biochemistry.

### Immunohistochemistry

For staining to be quantifiable and comparable, 4 brain sections were taken from positions 0.26, −0.46 −1.18 and −1.94 mm relative to bregma [36], from every mouse (n = 6–7), in all 8 groups and all 192 sections stained concurrently for each marker and developed for exactly the same period of time. Each marker used the next adjacent set of comparative sections from positions 0.26, −0.46 −1.18 and −1.94 mm relative to bregma. For brains, LAMP2, GFAP, VAMP2, and Isolectin B4 staining was performed as previously described [13], [16]. The anti-GM2 antibody (a gift from Dr Kostantin Dobrenis and Prof Walkley) was diluted 1/40 and the staining was performed as previously described [20]. Sections were visualised with diaminobenzidine (DAB substrate kit, Vector Labs Inc.). For quantification analysis, nickel was added to the DAB substrate to obtain black staining for easier quantification. Isolectin B4 and GFAP sections were counterstained with Mayer's haematoxylin. Liver sections were stained for LAMP2 as previously described [13].

### Image analysis

Two non-overlapping low power (x20 objective) fields of view were digitally photographed from each of the 4 sections, as shown in Figure 1A, using an Axioscope light microscope and Axiocam color CCD with Axiovision software (Figure 1A).

The first field of view was taken with the left edge of the field of view in line with the apex of the cingulum and at a right angle to the fibres of the corpus callosum and positioned so that cerebral cortical laminas II/III-V/VI were photographed. The second non-overlapping field of view was taken adjacent to the first field of view covering cerebral cortical laminas II/III-V/VI. This ensured that the same fields of view were taken for each section for each mouse (total 8 non-overlapping fields per mouse [n = 6–7/group]). For the hippocampus, two non-overlapping low power (x20 objective) fields of view were digitally photographed from the third and fourth sections (relative to Bregma −1.18 and 1.94) with the first field of view covering the CA1 region and the second covering the CA2 and CA3 regions of the hippocampus as shown in Figure 1A (total of 4 non-overlapping fields per mouse [n = 6–7/group]). Identical exposure settings were used for each stain and all photographs taken in one session. Images were transformed to eight bits of grey resolution and stored in TIFF format. To quantify LAMP2, VAMP2, GM2 staining, Image J software (NIH, USA) was used. Each entire unmanipulated field of view was blinded and quantified for the stain, whilst an unstained area was used to determine (and subtract) background staining for each section. An average of the levels of optical density for each section and each mouse was calculated. These were then averaged to give a group mean for cerebral cortex or hippocampus for each mouse. To quantify Isolectin B4 or GFAP staining, the number of positive cells per non-overlapping cortical field (as described above) was counted and all 8 fields averaged for each mouse. We have also included examples of full sized high quality images of GFAP staining that were used for counting astrocytes as supplementary data to show that individual astrocytes are easily distinguishable at this magnification (See Figures S1, S2, S3, and S4). These images correspond to the first field of view on section 2 as shown in Figure 1A, and to the images presented in Figure 2A.

### Biochemical assays

All samples were analyzed in blinded fashion. Liver samples were prepared as previously described. Total sulphated glycosaminoglycans in livers were measured using the Blyscan kit (Biocolor Ltd.,UK), standardized against DNA using PicoGreen ® dsDNA Kit (Invitrogen Ltd, UK) as previously described [13]. The final values were the mean of three tissue samples. High performance liquid chromatography (HPLC) was used to quantify the presence of pathological GAGs which have accumulated due to reduced enzyme activity in the brain from the second hemicoronal fifth (rostral to caudal) (SensiPro assay, Zacharon Pharmaceuticals Inc., data on file). Tissue GAG was extracted as previously described [37]. Samples were prepared by normalizing each to equivalent volumes using phosphate buffered saline followed by purification using reagents to extract impurities and isolate pathogenic GAG (pGAG). The pGAG were tagged with fluorescent dye, analyzed by HPLC [37] and this was standardized against total protein in each sample (Bradford assay). Tissue lipid extraction, isolation and quantitative densitometric studies of gangliosides after separation on silica gel 60 HPTLC plates were performed as previously described [23], [25].

### Statistics

For statistical analysis, extreme outliers in biochemical and immunohistochemical data were formally removed using the boxplot tool in SPSS (Those more than 3x the interquartile range outside of the end of the interquartile box). None were removed from behavioural data due to the possibility of excluding erratic phenotypes. Data were analysed using 3 way ANOVA for gender (Male/Female), genotype (MPSIIIB/WT), drug (Untreated/Genistein) and Tukey's multiple comparisons test used to determine differences between groups using JMP software (SAS Ltd, UK).

## Supporting Information

Figure S1 WT control GFAP stain x20.tif. The full sized TIFF image of GFAP (brown) stained cerebral cortex from an untreated 11 month old WT mouse. This image corresponds to the first field of view on section 2 as shown in Figure 1A, to the image presented in Figure 2A and was used to count the number of GFAP-positive cells. The section was counterstained with Mayer's haematoxylin (blue) to highlight the nuclei of cells.
(36.15 MB TIF)

Figure S2 MPS IIIB control GFAP stain x20.tif. The full sized TIFF image of GFAP (brown) stained cerebral cortex from an untreated 11 month old MPSIIIB mouse. This image corresponds to the first field of view on section 2 as shown in Figure 1A, to the image presented in Figure 2A and was used to count the number of GFAP-positive cells. The section was counterstained with Mayer's haematoxylin (blue) to highlight the nuclei of cells.
(36.15 MB TIF)

Figure S3 WT genistein treated GFAP stain x20.tif. The full sized TIFF image of GFAP (brown) stained cerebral cortex from a genistein treated 11 month old WT mouse. This image corresponds to the first field of view on section 2 as shown in Figure 1A, to the image presented in Figure 2A and was used to count the number of GFAP-positive cells. The section was counterstained with Mayer's haematoxylin (blue) to highlight the nuclei of cells.
(36.15 MB TIF)

Figure S4 MPS IIIB genistein treated GFAP stain x20.tif. The full sized TIFF image of GFAP (brown) stained cerebral cortex from a genistein treated 11 month old MPSIIIB mouse. This image corresponds to the first field of view on section 2 as shown in Figure 1A, to the image presented in Figure 2A and was used to count the number of GFAP-positive cells. The section was counterstained with Mayer's haematoxylin (blue) to highlight the nuclei of cells.
(36.15 MB TIF)
