# Supplementary figures and images for: Genistein Improves Neuropathology and Corrects Behaviour in a Mouse Model of Neurodegenerative Metabolic Disease
Source: PLoS One. 2010 Dec 1;5(12):e14192. doi: 10.1371/journal.pone.0014192 (PMC2995736; doi:10.1371/journal.pone.0014192)

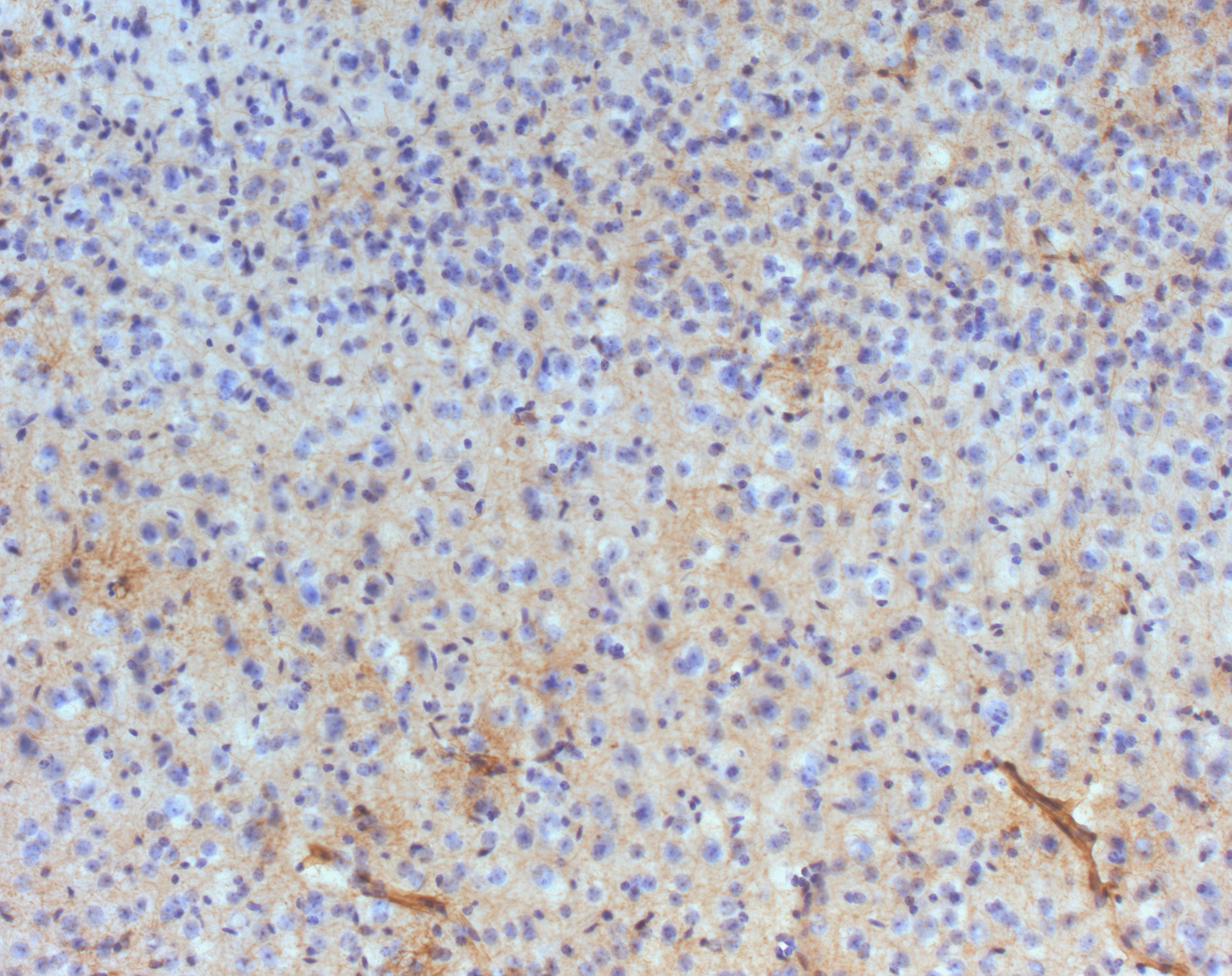

Supplement: Figure S1 — WT control GFAP stain x20.tif. The full sized TIFF image of GFAP (brown) stained cerebral cortex from an untreated 11 month old WT mouse. This image corresponds to the first field of view on section 2 as shown in Figure 1A, to the image presented in Figure 2A and was used to count the number of GFAP-positive cells. The section was counterstained with Mayer's haematoxylin (blue) to highlight the nuclei of cells. (36.15 MB TIF) [file pone.0014192.s001.tif]

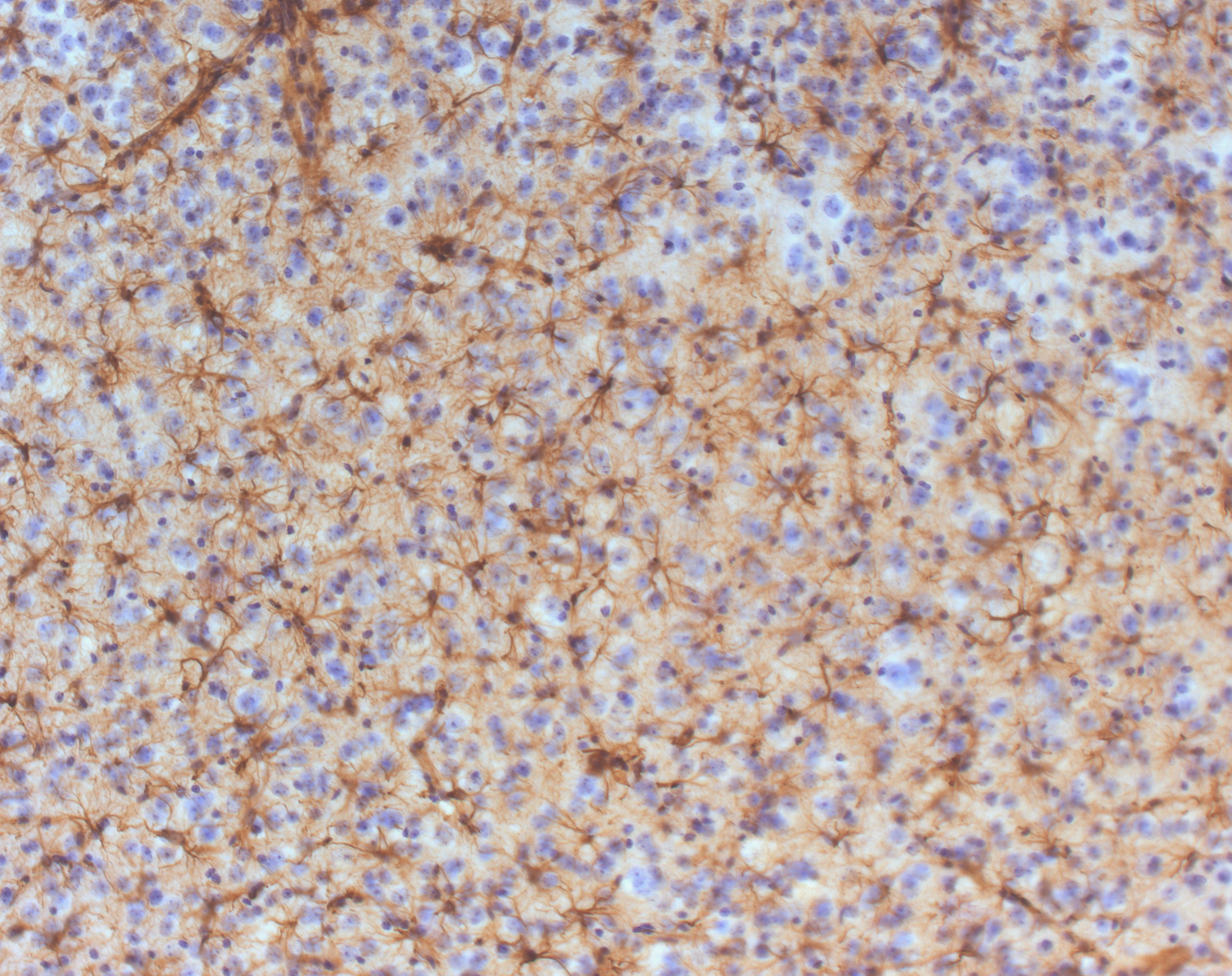

Supplement: Figure S2 — MPS IIIB control GFAP stain x20.tif. The full sized TIFF image of GFAP (brown) stained cerebral cortex from an untreated 11 month old MPSIIIB mouse. This image corresponds to the first field of view on section 2 as shown in Figure 1A, to the image presented in Figure 2A and was used to count the number of GFAP-positive cells. The section was counterstained with Mayer's haematoxylin (blue) to highlight the nuclei of cells. (36.15 MB TIF) [file pone.0014192.s002.tif]

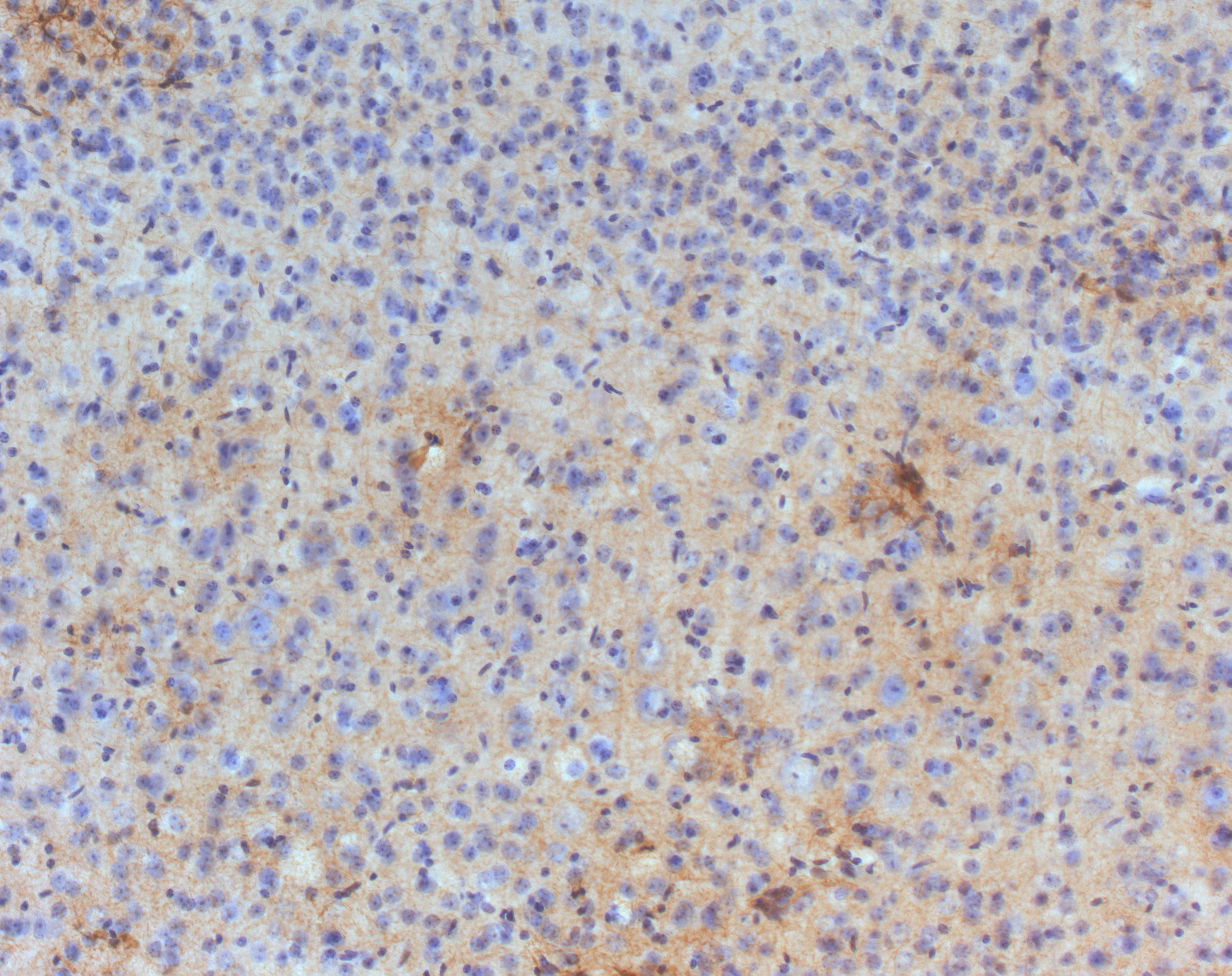

Supplement: Figure S3 — WT genistein treated GFAP stain x20.tif. The full sized TIFF image of GFAP (brown) stained cerebral cortex from a genistein treated 11 month old WT mouse. This image corresponds to the first field of view on section 2 as shown in Figure 1A, to the image presented in Figure 2A and was used to count the number of GFAP-positive cells. The section was counterstained with Mayer's haematoxylin (blue) to highlight the nuclei of cells. (36.15 MB TIF) [file pone.0014192.s003.tif]

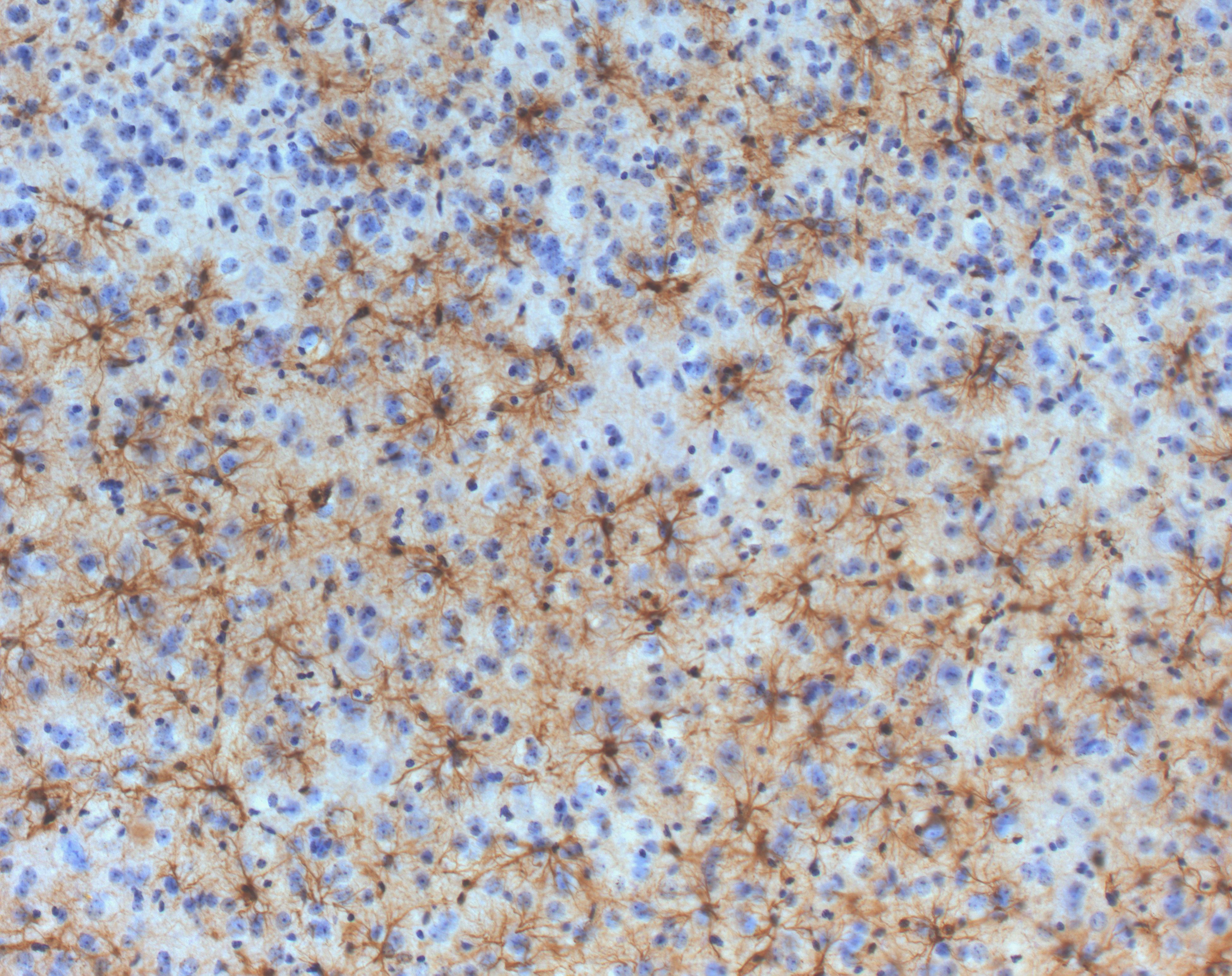

Supplement: Figure S4 — MPS IIIB genistein treated GFAP stain x20.tif. The full sized TIFF image of GFAP (brown) stained cerebral cortex from a genistein treated 11 month old MPSIIIB mouse. This image corresponds to the first field of view on section 2 as shown in Figure 1A, to the image presented in Figure 2A and was used to count the number of GFAP-positive cells. The section was counterstained with Mayer's haematoxylin (blue) to highlight the nuclei of cells. (36.15 MB TIF) [file pone.0014192.s004.tif]
